# Current Therapies in Kidney Transplant Rejection

**DOI:** 10.3390/jcm12154927

**Published:** 2023-07-27

**Authors:** Sami Alasfar, Lavanya Kodali, Carrie A. Schinstock

**Affiliations:** 1 Department of Medicine, Mayo Clinic, Phoenix, AZ 85054, USA; kodali.lavanya@mayo.edu; 2 Department of Medicine, Mayo Clinic, Rochester, MN 55905, USA; schinstock.carrie@mayo.edu

**Keywords:** rejection, transplantation, kidney transplant

## Abstract

Despite significant advancements in immunosuppressive therapies, kidney transplant rejection continues to pose a substantial challenge, impacting the long-term survival of grafts. This article provides an overview of the diagnosis, current therapies, and management strategies for acute T-cell-mediated rejection (TCMR) and antibody-mediated rejection (ABMR). TCMR is diagnosed through histological examination of kidney biopsy samples, which reveal the infiltration of mononuclear cells into the allograft tissue. Corticosteroids serve as the primary treatment for TCMR, while severe or steroid-resistant cases may require T-cell-depleting agents, like Thymoglobulin. ABMR occurs due to the binding of antibodies to graft endothelial cells. The most common treatment for ABMR is plasmapheresis, although its efficacy is still a subject of debate. Other current therapies, such as intravenous immunoglobulins, anti-CD20 antibodies, complement inhibitors, and proteasome inhibitors, are also utilized to varying degrees, but their efficacy remains questionable. Management decisions for ABMR depend on the timing of the rejection episode and the presence of chronic changes. In managing both TCMR and ABMR, it is crucial to optimize immunosuppression and address adherence. However, further research is needed to explore newer therapeutics and evaluate their efficacy.

## 1. Introduction

Kidney transplantation is considered the best treatment option for individuals with end-stage kidney disease (ESKD) [1]. Recent trends have shown an increase in the median survival of kidney allografts, and according to the United Network for Organ Sharing (UNOS), the one-year survival rate for kidney transplants is approximately 95%, while the five-year and ten-year survival rates are about 85% and 65%, respectively [2,3]. Despite advances in surgical techniques, immunosuppressive drugs, and improved patient management, kidney transplant rejection remains a significant issue that affects long-term graft survival [4]. Therefore, it is crucial to develop effective strategies to prevent and manage rejection in kidney transplant recipients to improve long-term graft survival and patient outcomes. Despite maximizing treatment of rejection episodes, some cases may not be reversed and may impact allograft survival. Even if maximum antirejection treatment is administered, certain kidney allografts may not recover function. Additionally, acute rejection episodes can negatively impact long-term graft survival for those who do recover [5].

Kidney transplant rejection can manifest as either subclinical or clinical. Subclinical rejection often lacks evident symptoms or laboratory abnormalities and is typically identified through protocol biopsy or biopsy for cause after observing an increase in surveillance biomarkers, such as donor-derived cell-free DNA or the detection of new donor-specific antibodies (DSAs). Surveillance methods for detecting subclinical rejection vary among centers, with some employing routine laboratory tests only, while others utilize a combination of protocol biopsies, DSA assessments, and biomarker monitoring. Consensus on the optimal surveillance approach for subclinical rejection has yet to be reached. Conversely, clinical rejection can manifest with a decline in kidney function, proteinuria, or hematuria, or it can be symptomatic and cause fever, allograft pain, hematuria, or decreased urine output, in addition to the laboratory abnormalities mentioned. The diagnosis of rejection typically requires assessing kidney allograft pathology, with findings being classified according to the Banff classification.

The Banff classification is a standardized system that grades and categorizes kidney transplant rejection based on histological findings. Initially established in 1991, the classification has undergone multiple revisions to enhance its accuracy and clinical relevance [6]. The Banff classification system categorizes rejection into acute T-cell-mediated rejection (aTCMR), chronic active T-cell-mediated rejection (caTCMR), active antibody-mediated rejection (aABMR), chronic active antibody-mediated rejection (caABMR), and chronic (inactive) antibody-mediated rejection (cABMR). However, it is common for patients to exhibit more than one type of rejection simultaneously. The system also includes specific criteria for borderline changes that do not meet the criteria for aTCMR.

The classification takes into account various acute and chronic histological features to diagnose rejection. The acute rejection changes include *i* (interstitial inflammation), *t* (tubulitis), *v* (arteritis), *g* (glomerulitis), PTC (peritubular capillary inflammation), and *c4d* (complement 4 d product), while chronic rejection changes include *ct* (tubular atrophy), *ci* (interstitial fibrosis), *cv* (arteriolopathy), *cg* (chronic glomerulopathy), and peritubular capillary multilayering. Most changes are typically diagnosed using light microscopy; however, *c4d*, a histologic marker of complement activation, is a distinctive histological feature that can be diagnosed using either immunofluorescence or immunohistochemistry. Additionally, peritubular capillary basement membrane multilayering can be visualized using electron microscopy [6,7]. Each change is graded from 0 to 3, and the severity of rejection is based on the severity of one or a combination of the lesions above.

## 2. T-Cell-Mediated Rejection (TCMR)

### 2.1. Diagnosis

The gold standard for diagnosing acute T-cell-mediated rejection is through histological examination of kidney biopsy samples. The biopsy samples typically reveal infiltration of mononuclear cells in the interstitium, tubules, and/or vessels (*t*, *i*, or *v*). caTCMR is characterized by chronic changes that include *ct*, *ci*, and/or *cv* lesions, in addition to acute inflammation changes (*t*, *i*, or *v*) [7]. The severity of aTCMR is graded into IA, IB, IIA, IIB, and III, which are outlined in Table 1 according to the Banff 2019 classification [7]. The majority of biopsy-proven acute rejections (BPARs) occurring within the first year post-transplant are typically TCMR [8].

### 2.2. Current Therapies

#### 2.2.1. Corticosteroids

Corticosteroids are the first-line treatment for acute inflammation in TCMR. Typically, a bolus dose of 3–5 mg/kg of intravenous (IV) methylprednisolone is administered over 3–6 consecutive days, followed by an oral prednisone taper. Corticosteroids act by inhibiting cytokine transcription by blocking transcription factors, such as NF-kβ and activator protein-1. This leads to downstream effects of T-cell depletion (by IL-2 inhibition), inhibition of T-Helper 1 differentiation and apoptosis, eosinophil apoptosis, and macrophage dysfunction [9].

The response to treatment for TCMR can vary based on the severity of the condition. Although the literature and guidelines may not explicitly define the criteria for response to therapy, it is generally understood that response to therapy in the context of TCMR is typically assessed by observing resolutions of histological findings and improvements in kidney function [10]. A study found that aTCMR Banff grade II had a decreased response to steroid therapy, resulting in poor allograft survival when compared to TCMR Banff grade IA. In this study, steroid therapy alone was able to reverse rejection in 36% of cases. However, 86% of the non-responders were successfully treated with anti-lymphocyte antibody therapy [11]. In a systematic review of literature aimed at assessing the response to treatment in TCMR, the authors observed that different criteria were employed across various studies for defining response to therapy. Despite the limitations posed by this heterogeneity in the criteria used, the pooled data revealed that the response to therapy was similar in Banff IA, IB rejections (44–73%) and Banff IIA rejections (52–80%), whereas the response was significantly lower in Banff IIB rejections (10%) [12].

#### 2.2.2. T-Cell-Depleting Agents

KDIGO recommends the use of T-cell-depleting agents in the treatment of steroid-resistant cellular rejection [13]. Thymoglobulin, which is a polyclonal agent containing antibodies to various antigens, is the most commonly used T-cell-depleting agent. It can interact with several receptors on T cells, as well as some shared receptors on B cells, monocytes, and neutrophils. The primary mechanism of action is lymphocyte depletion through complement-dependent lysis and induction of T-cell activation-induced apoptosis [14]. There are two preparations available: Thymoglobulin, made by immunizing rabbits with human thymocytes, and anti-thymocyte globulin (rATG), made by immunizing rabbits with lymphocytes from the Jurkat T-cell leukemia line. Both products induce cytokine release due to their non-human immunoglobulins by activation of NK cells and macrocytes/monocytes binding to the Fc receptor, as well as cellular cytotoxicity. Thymoglobulin is generally used at a dose of 1–1.5 mg/kg per dose for a total of 4–6 doses [15]. Leukopenia and thrombocytopenia are commonly encountered. Peripheral CD3 counts can be used to monitor response. In patients who develop thymoglobulin antibodies, ATGAM (horse anti-thymocyte globulin) can be used at a dose of 1 gm IV for 10 days, with further dosing adjusted based on peripheral CD2-positive T-cell measurement. However, the use of ATGAM is associated with significant side effects, including serum sickness and cytokine release, and it is less well tolerated than thymoglobulin.

### 2.3. Approach to Treatment

**Figure 1:**
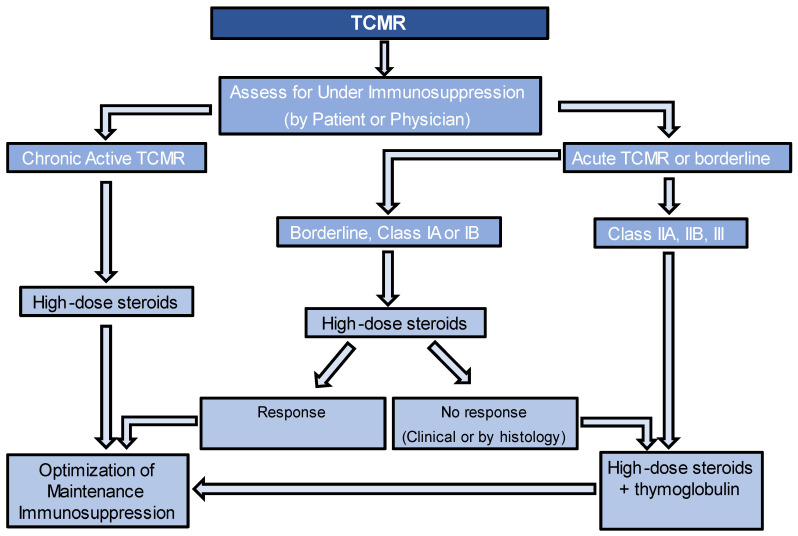
Algorithm for management of T-Cell-Mediated Rejection.

TCMR can be the clinical diagnosis via for-cause biopsy or the subclinical diagnosis through protocol biopsies. Although there are currently no established guidelines on how to treat clinical versus subclinical TCMR, most transplant nephrologists would recommend treatment of both entities, as long as there are no contraindications [16]. The approach to managing TCMR can vary among transplant centers. For TCMR IA and IB, despite lack of data showing their beneficial impact on kidney transplant rejection outcomes, high-dose corticosteroids are typically used, while thymoglobulin is usually utilized for grade II or III TCMR or if TCMR IA or IB does not respond to treatment. Some centers may perform follow-up biopsies after treatment to assess the response to therapy. While there is no consensus on the exact timing of these biopsies, it is typically performed 2–4 weeks after completing the therapy. Figure 1 provides an algorithm for managing TCMR. For caTCMR, high-dose steroids alone are usually considered. Also, it is important to check for the presence of DeNovo DSA at the time of diagnosis, even in the absence of histological findings of ABMR. Regardless of the type of TCMR, it is important to optimize maintenance immunosuppression, unless there are contraindications. This may involve targeting higher levels of tacrolimus or cyclosporine, adding prednisone if it is not already being used, and ensuring patients are on the highest tolerated dose of mycophenolate up to 2000 mg per day. If patients are not already taking mycophenolate, it should be added to their regimen or switched from azathioprine, if that is currently being used. Another key point to consider is the evaluation of nonadherence, as it is important to recognize that if nonadherence is identified, increasing the targets of immunosuppression may not be necessary. The guidelines for monitoring the response to therapy in TCMR lack consensus. However, various approaches can be employed, either individually or in combination, to assess the response. These include follow-up kidney function tests and follow-up biopsies.

## 3. Borderline Changes

Borderline changes in kidney transplant rejection refer to a spectrum of histological findings that lie between normal graft histology and acute cellular rejection (Table 1). Borderline changes are often characterized by mild inflammation, scattered infiltrating immune cells, and subtle signs of tissue damage. Although these changes do not meet the strict criteria for acute cellular rejection, they are considered significant because they indicate an ongoing immunological response against the transplanted organ.

The management of borderline changes typically involves optimizing immunosuppressive therapy if there are no contraindications. Additionally, most centers typically choose to address borderline rejection by administering a course of oral or IV steroids. This is due to the proven negative outcomes of borderline changes. For example, in a prospective study comparing the outcomes of patients with subclinical inflammation (Banff < 1A, *n* = 129) to those without inflammation (*i + t* = 0, *n* = 71) on a 3-month protocol biopsy showed that the group with subclinical inflammation had higher serum creatinine levels (*p* = 0.02) and chronicity scores on the follow-up biopsy at 12 months (*ci*, *ct*, *cv*, *cg*) (*p* = 0.02), and a greater likelihood of developing de novo DSA and experiencing rejection episodes requiring treatment during the two-year follow-up period [17].

## 4. Antibody-Mediated Rejection (ABMR)

### 4.1. Diagnosis

ABMR is caused by the binding of antibodies circulating in the recipient’s blood to donor alloantigens on graft endothelial cells [18]. The most common alloantigens involved in ABMR are human leukocyte antigen (HLA) class I and class II antigens, as well as ABO blood group antigens in recipients of ABO-incompatible transplants. In addition to these major histocompatibility complex (MHC) alloantigens, other antigens on the endothelium may also be targeted, such as Angiotensin II type 1 receptor, endothelin receptor A, and MHC class I chain-related gene A (MICA) antibodies [19,20,21]. Criteria for diagnosis of aABMR are listed in Table 2 [5]. caABMR is a pathological process that occurs due to ongoing antibody damage, leading to chronic endothelial cell injury and remodeling of the allograft matrix [22]. caABMR diagnostic criteria are similar to those of aABMR, but add the presence of chronic changes of *cg*, peritubular capillaries basement membrane multilayering, or chronic arteriolar intimal thickening *cv*. Chronic (inactive) caABMR is characterized by the presence of these chronic changes without active inflammation. In addition to the phenotypes of ABMR mentioned in the table, the Banff 2022 meeting introduced two additional subtypes. These include probable ABMR, characterized by the presence of circulating DSA and individual lesions of microvascular inflammation (MVI) that fall below the histological threshold for MVI (g + ptc < 2). The second subtype is MVI, which surpasses the histological threshold, but lacks circulating DSA and exhibits negative C4d staining in peritubular capillaries [23].

### 4.2. Current Therapies

#### 4.2.1. Plasmapheresis

Plasmapheresis, or plasma exchange, is the most common treatment for ABMR. This technique has been used since 1979 in patients with ABMR. Plasmapheresis directly removes DSAs from the serum in a predictable manner. However, since DSAs are only removed from the vascular compartment, there is re-equilibration with the interstitium over time, increasing the DSA levels in the serum again. As this equilibrium process takes about 48 h, plasmapheresis is usually spaced every 48 h, although it could be conducted consecutively for the first few days after diagnosis of ABMR to rapidly remove existing antibodies [24]. Studies have suggested that plasmapheresis has an immuno-modulatory effect, reducing B cells and NK cells, and increasing T regulatory cells and T-suppressor-cell function [25,26,27].

A randomized controlled study by Bonomini et al. showed a significant beneficial effect of plasmapheresis in steroid-resistant, DSA-positive rejection (30% vs. 81% graft loss) when compared to the control group [28]. However, a randomized controlled trial of 27 patients with ABMR unresponsive to high-dose steroids did not show any benefit in graft survival using plasma exchange therapy. The median graft survival was 12 days for the treatment group with plasmapheresis and 25 days for the controls [24]. While some studies have shown a benefit of plasmapheresis in the treatment of ABMR, others have not [29,30,31,32,33]. It is worth noting that the inclusion criteria, primary endpoints, and duration of follow-up varied considerably among these studies. Furthermore, the lack of success observed in some older trials may be attributed to the use of different eras of immunosuppression, as current immunosuppressive therapies are known to be more effective and safer than those employed at that time.

#### 4.2.2. Immunoadsorption

Immunoadsorption (IA) is another method that works by directly removing IgG proteins from the serum using high-affinity absorbers in adsorption columns. A randomized controlled trial by Bohmig et al. compared the efficacy of IA to conventional triple immunotherapy with CNI, mycophenolate, and steroids in the treatment of severe ABMR. Five patients were randomly assigned to each group, and all patients in the IA group were on dialysis compared to four out of five patients in the control group. All patients treated with IA responded to treatment at 3 weeks, while the four control subjects continued to remain on dialysis. The study was terminated early due to high graft loss in the control group, suggesting the effective role of IA in reversing severe C4d-positive AMR [34,35]. There is currently a lack of high-quality trials comparing the response between IA and plasmapheresis. However, in a retrospective study, it was observed that the 5-year graft survival showed a positive trend in the IA group (*n* = 17) compared to the plasmapheresis group (*n* = 30). IA therapy is currently not available in the United States [36].

#### 4.2.3. Intravenous Immunoglobulin (IVIG)

IVIG is commonly used in conjunction with plasmapheresis for managing ABMR, with variable dosages across centers. Low-dose IVIG (100 milligram/km) or high-dose IVIG (2 g/km) with corticosteroids are the commonly used regimens, with low-dose IVIG inhibiting endogenous antibody rebound after plasmapheresis removal and restoring protective antimicrobial IgGs [37]. At high doses, IVIG has immunomodulatory effects on T and B cells, inducing B-cell apoptosis and modulating B-cell signaling [38]. To determine if IVIG treatment in late ABMR (>6 months after transplantation) improves allograft outcomes, Lee et al. performed a retrospective study and found that patients who received IVIG had improved graft survival compared to those who did not [39]. Patients with C4d-positive ABMR on biopsy were divided into two groups—those who received IVIG (intervention group) versus those who did not (control group). Four sessions of plasmapheresis every other day followed by IVIG 0.5 g/kg was given in the intervention group. With a mean follow-up of 7 years, improved graft survival was observed in the intervention group compared to the controls (HR 0.26; *p* < 0.001). It should be noted that about 50% of patients in this study had chronic lesions on histology. In a small retrospective study, high-dose IVIG was compared to plasmapheresis/IVIG/anti-CD20 therapy in the treatment of biopsy-proven ABMR, with graft survival being higher in the PLEX/IVIG/anti-CD20 group. Graft survival was 50% in the IVIG group vs. 91.7% in the PLEX/IVIG/anti-CD20 group (*p* = 0.02) at 36 months [40]. Due to the heterogeneity of previous studies, with variations in doses, biopsy findings, time from transplantation, and maintenance immunosuppression, it is difficult to compare the studies [41,42,43,44].

#### 4.2.4. Anti-CD20 Monoclonal Antibody

Rituximab is sometimes used to treat ABMR, and the KDIGO guidelines recommend (grade 2C) its use as a treatment option [13]. Rituximab works by depleting B cells, which are precursors of plasma cells that produce DSA. However, there is a lack of consensus on the optimal dosing regimen for rituximab. Side effects include infusion reactions, lymphopenia, neutropenia, infections, and development of Progressive Multifocal Leukoencephalopathy. Reactivation of hepatitis B with fulminant hepatitis, hepatic failure, and death can occur, so patients should be screened for hepatitis B before starting rituximab [45].

Despite its frequent use, there is limited evidence to support its effectiveness. The RITUX-ERAH study examined the effectiveness of rituximab in 38 patients with aABMR treated with plasmapheresis and IVIG, who were randomly assigned to receive either rituximab or a placebo. The study found no significant differences in outcomes between the two groups, except for side effects, which were more common in the rituximab group [46]. In another prospective study, which evaluated the effectiveness and safety of IVIG combined with or without rituximab in caABMR, the study found no significant differences in outcomes between the treatment and placebo groups [47].

Retrospective studies have shown some benefit of rituximab in treating ABMR. One study, which administered rituximab alongside other therapies, resulted in successful treatment in 24 out of 27 patients [48]. Another study that evaluated 54 patients with ABMR found that those who received plasmapheresis plus rituximab had a significantly higher two-year graft survival rate compared to those who only received plasmapheresis (90% vs. 60%) [49]. However, systematic reviews have failed to provide clear evidence for the effectiveness of rituximab in improving ABMR outcomes [50].

#### 4.2.5. Complement Inhibitors

The activation of the classical pathway of the complement system is responsible for the downstream effects of DSA, and is a significant factor in the manifestations of ABMR [51]. Eculizumab, a monoclonal antibody that targets complement 5, and complement 1 esterase inhibitors (C1 INHs) are examples of complement inhibitors that have been utilized for the prevention and treatment of AMR, with varying degrees of effectiveness.

Eculizumab functions by blocking the activation of the complement system, specifically targeting complement protein C5. Potential side effects of eculizumab treatment include infusion reactions, as well as an increased risk of infections with encapsulated bacteria, such as pneumococcus, haemophilus, and neisseria [52]. Therefore, patients receiving it should receive necessary vaccines against these pathogens and/or receive prophylactic antibiotics.

Several case reports have described successful use of eculizumab in severe or refractory cases of ABMR in HLA- or ABO-incompatible kidney transplants [53,54,55,56,57]. Orandi et al. conducted a retrospective comparison of the combination of splenectomy with eculizumab to either splenectomy alone or eculizumab alone as an add-on therapy to plasmapheresis/IVIG and rituximab for the treatment of early ABMR in HLA-incompatible kidney transplant recipients. The combined strategy was found to be the most effective, with no graft loss compared to the splenectomy-alone group and the eculizumab-alone group [58].

In the only randomized controlled trial assessing eculizumab efficacy for treatment of caABMR, eculizumab for 6 months was compared with observation. There was no difference between treatment groups, but the eculizumab group did have an improved trajectory of renal function in the treatment group [59]. Overall, and in spite of several case reports that described the use of eculizumab for this indication, the current available literature does not provide enough evidence for the routine use of eculizumab in the treatment of AMR. The potential benefit of complement in personalized treatment approaches, such as ABMR with C1q fixing DSA or C4d-positive ABMR, requires further investigation and evidence.

C1 INH has been studied in the treatment for ABMR [51]. In a pilot study evaluating the use of C1 INH for the treatment of ABMR nonresponsive to standard treatment for 3 months, six patients showed improvement in mean eGFR after 6 months of treatment [60]. Although mean DSA levels remained stable, there was a significant decline in the anti-HLA DSA C1q status (6/6 to 1/6 at 6 months, *p* = 0.025). A phase 2b, multicenter, double-blind, randomized, placebo-controlled pilot study also assessed the use of Cinryze, a human plasma-derived C1 INH, as add-on therapy for ABMR occurring within the first year after transplant [61]. The primary endpoint of day 20 pathology or graft survival was not significantly different between the two groups. However, six-month protocol biopsies showed that none of the patients in the C1 INH group had TG (CG ≥ 1b), whereas three of the seven placebo subjects did. It is important to note that a larger study evaluating the efficacy and safety of Cinryze for the treatment of ABMR was terminated early in 2019 due to futility [51].

#### 4.2.6. Proteasome Inhibitors

Bortezomib, a proteasome inhibitor commonly used in the management of multiple myeloma, has also been utilized in the treatment of ABMR. Bortezomib’s effectiveness against differentiated plasma cells is due to their elevated protein synthesis rate, which bortezomib targets and induces plasma cell apoptosis [62]. It was first used in 2008, and several case reports indicated its efficacy in ABMR when given with other therapies [62,63]. Side effects include gastrointestinal symptoms, peripheral neuropathy, and thrombocytopenia [64].

Bortezomib has demonstrated efficacy in retrospective studies [65,66,67,68]. In the only trial (BORTEJECT Trial), which investigated whether bortezomib could prevent GFR decline in late DSA-positive ABMR in kidney transplant recipients, the randomized, placebo-controlled trial found that bortezomib did not significantly improve the eGFR slope, 2-year graft survival, urinary protein concentration, DSA levels, or rejection phenotypes compared to placebo, and was associated with significant gastrointestinal and hematologic toxicity [69].

#### 4.2.7. Splenectomy

Although surgical splenectomy is not routinely performed in patients with ABMR, some centers consider it as a salvage procedure for cases refractory to plasmapheresis and/or IVIG. In a case series of four patients with severe refectory ABMR, laparoscopic splenectomy resulted in immediate improvement in the urine output and a decrease in serum creatinine within 48 h [70]. Similarly, in another case series of five patients who underwent living-donor kidney transplantation after desensitization and subsequently developed ABMR, splenectomy followed by plasmapheresis and IVIG led to the return of allograft function within 48 h of the procedure [71]. However, given the lack of evidence supporting the safety and efficacy of splenectomy over medical therapy, it is not routinely recommended [18].

### 4.3. Approach to Treatment

**Figure 2:**
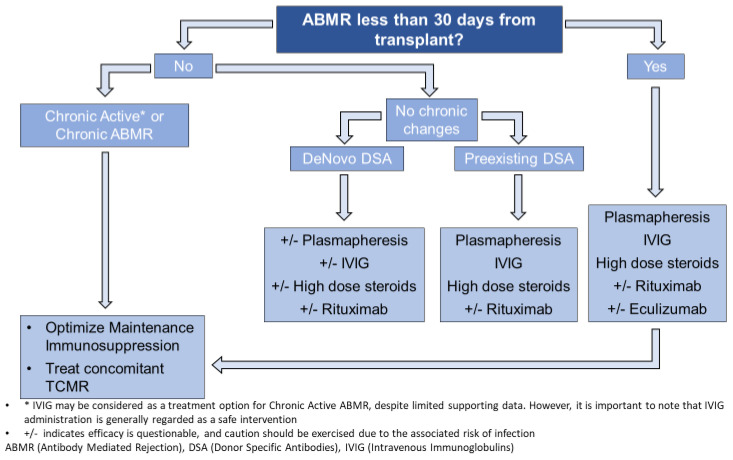
Algorithm for management of Antibody-Mediated Rejection.

Similar to TCMR, ABMR can be diagnosed clinically via a for-cause biopsy or subclinically via a protocol biopsy. The management of ABMR, whether clinical or subclinical, varies among transplant centers and often involves a combination of therapies, typically including high-dose steroids, plasmapheresis, and IVIG. However, there is significant variation in the use of other therapies. The transplant society has established consensus guidelines that we are presenting in this approach. The timing and presence of chronic changes in ABMR holds significant importance in the management decision. It is worth noting that plasmapheresis, although occasionally considered, does not directly target the underlying cause of ABMR and should be approached sparingly [18]. According to a systematic review published in 2018, despite the evidence uncertainty, plasmapheresis and IVIG have become standard-of-care for the treatment of ABMR. Rituximab is not effective, and there is uncertainty about the role of bortezomib and complement inhibitors [50]. Figure 2 is an algorithm that incorporates both the consensus recommendations and our own clinical experience.

Early rejections tend to respond more positively to treatment, and medical centers frequently take an assertive approach in such cases. Early rejections are usually due to a rebound in pre-existing DSAs in sensitized patients, and less commonly, by DeNovo DSA that has not developed over a long enough period to cause irreversible damage.

If ABMR occurs later in the course of transplant, it is important to assess the presence of chronic changes, which usually indicate a poor prognosis, as aggressive therapies have not been shown to halt the progression of such changes. Additionally, it is also important to identify the type of DSA present. DeNovo DSAs can be more challenging to eradicate compared to preexisting DSAs, possibly due to the fact that patients with a preexisting DSA are typically monitored more closely. Given the challenges of managing late ABMR and the limited evidence available, an individualized approach should be taken. The potential benefits of different therapies should be weighed against the risks of infection and malignancy.

Similar to TCMR, in both early and late cases of ABMR, it is important to optimize maintenance immunosuppression, unless there are contraindications. In addition, ensuring patients adhere to their regimen and helping them overcome barriers to adherence is essential, as non-adherence is a common cause of ABMR [18,72,73].

## 5. Conclusions

In summary, rejection remains a significant concern in kidney transplantation and has a significant impact on the long-term outcomes of transplantation. The treatment approach varies depending on the type of rejection, whether it is T-cell-mediated rejection or antibody-mediated rejection. The Banff classification system is commonly used to diagnose and distinguish different types of rejection based on histological findings. For TCMR, treatment options typically involve the use of corticosteroids and T-cell-depleting agents, such as thymoglobulin. The severity of TCMR determines the specific treatment approach. In the case of ABMR, rejection occurs due to the binding of antibodies to graft endothelial cells. Managing ABMR typically involves a combination of therapies, such as corticosteroids, plasmapheresis, IVIG, and other adjunctive therapies. It is important to acknowledge that the efficacy of many of these therapies may be questionable. In all instances of rejection, optimizing maintenance immunosuppression is crucial to prevent further rejection episodes. Additionally, emerging therapies, such as IL-6 inhibitors; newer plasma cell inhibitors, such as carfilzomib; and Daratumumab show promise and will be discussed in another article in this issue. Since rejection is controlled by multiple immunological pathways, a likely combination of therapies is often necessary to reverse rejection. However, the use of these therapies should be carefully conducted through randomized controlled trials to ensure that the potential increased risk of infection is balanced with the benefits.

## Figures and Tables

**Table 1:** Summary of TCMR and borderline changes classification and pathological findings.

Class/Grade of Rejection		Minimal Pathological Findings Required for Diagnosis
aTCMR ^α^	Grade IA	*t*2 + (*i*2 or *i*3)
	Grade IB	*t3* + (*i*2 or *i*3)
	Grade IIA	*v*1 with or without interstitial inflammation and/or tubulitis
	Grade IIB	*v*2 with or without interstitial inflammation and/or tubulitis
	Grade III	*v*3 with or without interstitial inflammation and/or tubulitis
caTCMR ^β^	Grade IA	interstitial inflammation that affects more than 25% of sclerotic cortical parenchyma and more than 25% of the total cortical parenchyma (*ti*2 or *ti*3) with *t*2
	Grade IB	interstitial inflammation that affects more than 25% of sclerotic cortical parenchyma AND more than 25% of the total cortical parenchyma (*ti*2 or *ti*3) AND *t*3
	Grade II	Chronic allograft arteriopathy (arterial intimal fibrosis with mononuclear cell inflammation in fibrosis and formation of neointima)
Borderline Changes		*i*1, with *t*1, *t*2, or *t*3 or *t*1 with *i*2 or *i*3

^α^ aTCMR: Acute T-cell-mediated rejection; ^β^ caTCMR: Chronic active T-cell-mediated rejection.

**Table 2:** Criteria for diagnosis of active ABMR (all 3 criteria must be met).

Criteria 1	Histologic evidence of acute tissue injury, including 1 or more of the following: Microvascular inflammation (g > 0 and/or ptc > 0), in the absence of recurrent or de novo glomerulonephritis Intimal or transmural arteritis (v > 0) Acute thrombotic microangiopathy, in the absence of any other cause Acute tubular injury, in the absence of any other apparent cause
Criteria 2	Evidence of current/recent antibody interaction with vascular endothelium, including 1 or more of the following: Linear C4d staining in peritubular capillaries or medullary vasa recta At least moderate microvascular inflammation ([g + ptc] ≥ 2), in the absence of recurrent or de novo glomerulonephritis Increased expression of gene transcripts/classifiers in the biopsy tissue strongly associated with ABMR, if thoroughly validated
Criteria 3	Serologic evidence of circulating donor-specific antibodies (DSA to HLA or other antigens); C4d staining or expression of validated transcripts/classifiers as noted above in criterion 2 may substitute for DSA
